# Preparing Nurses for Lifestyle‐Focused Cardiovascular Care

**DOI:** 10.1002/nop2.70653

**Published:** 2026-06-18

**Authors:** Brenda Owusu, Priscilla Bivins, Kayla Baptiste, Serina Gbaba

**Affiliations:** ^1^ University of Miami School of Nursing & Health Studies Coral Gables Florida USA; ^2^ Broward College of Nursing Davie Florida USA; ^3^ University of South Florida College of Nursing Tampa Florida USA; ^4^ Meritus Health Hagerstown Maryland USA

## 1 Introduction

Cardiometabolic diseases remain the leading drivers of morbidity and mortality globally, despite advances in clinical care (World Health Organization [WHO] 2024; Martin et al. 2024). In the United States, the disease burden continues to rise, fuelled by ageing populations, persistent health inequities and the growing prevalence of modifiable risk factors such as poor diet, physical inactivity, obesity and chronic stress (Centers for Disease Control and Prevention 2024). Primary prevention policies for cardiometabolic conditions are inconsistently implemented across the continuum of care. Without a stronger emphasis on prevention, the clinical and economic burden of these conditions will continue to escalate. Cardiovascular disease‐related mortality in the US has not decreased since 2010 (Reddy and Freeman 2022) despite technological and research advances in recent decades. Additionally, disparities in cardiometabolic outcomes persist across racial, socioeconomic, and geographic lines, reflecting broader structural inequities in access to prevention and care. These patterns reflect not only individual risk but also systemic gaps in prevention delivery across care settings. Without targeted prevention strategies, these gaps are likely to persist and widen. This editorial argues that preparing nurses to deliver behaviour change and lifestyle‐focused interventions is an urgent and underdeveloped priority in cardiovascular care, requiring immediate alignment of nursing education and practice with prevention‐focused models.

## 2 The Shift to Lifestyle‐Focused Care

There is an evolving trend towards lifestyle medicine in cardiovascular care. Lifestyle medicine emphasizes long‐term behaviour modification rather than short‐term results. Clinical guidelines have increasingly shifted from a disease‐treatment paradigm towards primary and secondary prevention through a behaviour‐centred model of care. Frameworks such as the American Heart Association Life's Essential 8 highlight the central role of lifestyle behaviours, including nutrition, physical activity, sleep, and tobacco avoidance, in determining cardiovascular outcomes (Reddy and Freeman 2022; Martin et al. 2024). A critical shift in cardiovascular disease management is prevention and long‐term risk reduction through lifestyle modification, becoming a core goal of care. This development reflects a growing recognition that sustained behavioural change is fundamental to improving long‐term cardiovascular health. Disease prevention is no longer considered optional but viewed as central to managing cardiovascular diseases. Consequently, lifestyle medicine should be more consistently integrated as a foundational component of cardiovascular care, alongside pharmacologic management.

## 3 The Gap in Practice and Preparation

Cardiovascular diseases are often shaped by long‐term behavioural and social determinants of health. Behaviour change counselling, nutrition, and lifestyle‐based interventions may lead to decreasing the incidence of cardiovascular diseases and promoting health outcomes. Evidence suggests that nursing education has historically emphasised disease management over behaviour change competencies, limiting preparedness for prevention‐focused care (Creighton et al. 2025). Although nurses are highly skilled in caring for patients with cardiovascular disease, currently, there is a gap in their education as it relates to preventative interventions. Nursing students are not always taught how to deal with the specifics of behaviour modification.

Despite this trend towards lifestyle medicine in cardiovascular care, healthcare systems have not fully aligned with this model. Time constraints, fragmented care models, and limited training in behaviour change counselling continue to impede the consistent delivery of lifestyle‐focused interventions. This gap in implementation is closely linked to how the nursing workforce is prepared to deliver prevention‐focused care. Gaps in preparation remain in areas essential to prevention‐focused cardiovascular care. Early‐career nurses and nurse practitioners often have limited training in behaviour change counselling, nutrition, and lifestyle‐based interventions. While clinical management is emphasized, less attention is given to addressing the behavioural drivers of disease. In many settings, prevention remains constrained by visit structures and reimbursement models that prioritize acute management over sustained behaviour change. Addressing these gaps requires a workforce capable of delivering prevention‐focused care. Table 1 outlines the major educational competencies and curricular considerations necessary for lifestyle‐focused cardiovascular care.

**TABLE 1:** Gaps in nursing preparation and strategies for prevention‐focused cardiovascular care.

Gap in preparation	What this looks like in practice	Recommended strategy
Limited training in behaviour change counselling	Nurses may feel uncertain about guiding patients in making and sustaining lifestyle changes	Integrate motivational interviewing and behaviour change training into nursing curricula
Emphasis on treatment over prevention	Care is often focused on managing disease rather than preventing it	Shift education and clinical training to prioritize prevention and risk reduction
Limited exposure to lifestyle‐based care	Few opportunities to practice nutrition and lifestyle counselling in clinical settings	Increase clinical experiences that include lifestyle‐focused interventions
Variability across nursing programmes	Inconsistent coverage of prevention and lifestyle medicine content	Establish clearer expectations for prevention‐focused competencies in BSN and MSN programmes
Limited access to experienced preceptors	Students may lack mentorship in applying prevention strategies in real‐world settings	Strengthening preceptor networks with expertise in cardiovascular prevention
Time and system constraints	Clinical visits may not allow time for prevention‐focused counselling	Support care models that allow time for patient education and behaviour change
Need for ongoing skill development	Limited opportunities to build prevention‐related skills after graduation	Expand continuing education in lifestyle medicine and behaviour change strategies

## 4 Nursing as the Solution

Within this gap lies a critical opportunity and responsibility for nursing. Nurses are uniquely positioned to lead prevention efforts through patient education, care coordination, and sustained engagement, serving as essential drivers of lifestyle‐focused cardiovascular care. Evidence demonstrates that nurse‐led interventions improve cardiovascular risk factor control, medication adherence and patient self‐management behaviours (Martin et al. 2024). Their continuous presence across care settings positions them to reinforce behaviour change over time, an essential component of effective prevention that is often missing from episodic care models. Prior work has also highlighted the importance of health literacy and patient engagement in managing chronic conditions, reinforcing the need for nurses to support behaviour change and prevention‐focused care (Owusu et al. 2024).

Nurses are also well‐positioned to advance lifestyle medicine as a core component of care, particularly through patient education and behaviour change support. Professional organizations such as the Preventive Cardiovascular Nurses Association (PCNA) can further support these efforts by promoting lifestyle‐focused, prevention‐oriented practice (Pike et al. 2025).

## 5 Call to Action

Cardiovascular prevention cannot be fully realized without a nursing workforce equipped to deliver it. Addressing this gap requires a workforce capable of delivering prevention‐focused care. Therefore, nursing educators must adjust their curriculum to emphasize the practice of preventative interventions. Integrating behaviour change training, strengthening clinical mentorship, and expanding access to continuing education are essential steps in preparing nurses to meet these demands. Professional organizations such as the Preventive Cardiovascular Nurses Association can play a key role in advancing lifestyle medicine and prevention‐focused training within BSN and MSN programmes. This shift also requires alignment at the system level, including support for prevention‐focused care delivery and recognition of behaviour change counselling as a core clinical competency. Without these structural changes, efforts to integrate lifestyle‐focused care into practice will remain limited. Nurses are not only central to patient care but also to prevention. Supporting workforce preparation with the realities of modern cardiovascular care is not optional; it is a public health imperative. In nursing practice, this requires expanding the role of nurses beyond disease management to include the structured delivery of behaviour‐change and lifestyle‐focused interventions, integrated early in the nursing curriculum.

## 6 Conclusion

As the focus of care continues to shift towards lifestyle modification and long‐term risk reduction, nursing practice and education must evolve accordingly. This shift has clear implications for nursing practice, education, and leadership in cardiovascular disease prevention. Preparing nurses to deliver lifestyle‐focused, prevention‐oriented care is no longer an aspirational goal; it is a necessary response to the growing burden of cardiometabolic disease. Aligning nursing education and practice with prevention‐focused care is essential to ensuring nurses are equipped to address behavioural drivers and ameliorate cardiovascular disease care. Without these changes, the gap between prevention goals and clinical practice will persist.

## Funding

The authors have nothing to report.

## Conflicts of Interest

The authors declare no conflicts of interest.

## Data Availability

Data sharing not applicable to this article as no datasets were generated or analysed during the current study.
